# Up-regulation of the human-specific CHRFAM7A gene in inflammatory bowel disease

**DOI:** 10.1016/j.bbacli.2015.12.003

**Published:** 2016-01-08

**Authors:** Andrew Baird, Raul Coimbra, Xitong Dang, Brian P. Eliceiri, Todd W. Costantini

**Affiliations:** a Division of Trauma, Surgical Critical Care, Burns and Acute Care Surgery, Department of Surgery, University of California San Diego, La Jolla, CA, USA; b The Key Laboratory of Medical Electrophysiology, Institute of Cardiovascular Research, Sichuan Medical University, Luzhou, China

## Abstract

Background: The α7-subunit of the α7-nicotinic acetylcholine receptor (α7-nAChR) is an obligatory intermediate for the anti-inflammatory effects of the vagus nerve. But in humans, there exists a second gene called CHRFAM7A that encodes a dominant negative α7-nAChR inhibitor. Here, we investigated whether their expression was altered in inflammatory bowel disease (IBD) and colon cancer.

Methods: Quantitative RT-PCR measured gene expression of human α7-nAChR gene (CHRNA7), CHRFAM7A, TBC3D1, and actin in biopsies of normal large and small intestine, and compared to their expression in biopsies of ulcerative colitis, Crohn's disease, and colon cancer.

Results: qRT-PCR showed that CHRFAM7A and CHRNA7 gene expression was significantly (p < .02) up-regulated in IBD (N = 64). Gene expression was unchanged in colon cancer. Further analyses revealed that there were differences in ulcerative colitis and Crohn's Disease. Colon biopsies of ulcerative colitis (N = 33) confirmed increased expression of CHRFAM7A and decreased in CHRNA7 expression (p < 0.001). Biopsies of Crohn's disease (N = 31), however, showed only small changes in CHRFAM7A expression (p < 0.04) and no change in CHRNA7. When segregated by tissue source, both CHRFAM7A up-regulation (p < 0.02) and CHRNA7 down-regulation (p < 0.001) were measured in colon, but not in small intestine.

Conclusion: The human-specific CHRFAM7A gene is up-regulated, and its target, CHRNA7, down-regulated, in IBD. Differences between ulcerative colitis and Crohn's disease tie to location of disease.

Significance: The appearance of IBD in modern humans may be consequent to the emergence of CHRFAM7A, a human-specific α7-nAChR antagonist. CHRFAM7A could present a new, unrecognized target for development of IBD therapeutics.

## Highlights

• CHRFAM7A is a pro-inflammatory and human-specific gene not found in other species.
• CHRFAM7A expression is elevated in certain IBD, but its target CHRNA7 decreased.
• Changes in CHRFAM7A and CHRNA7 expression are disease- and tissue site specific.
• Some IBDs may be examples of “off-target disease sequelae” of human evolution.
• Animal modeling of human disease do not test contributions of human-specific genes.

## 1 Introduction

The emergence of human-specific genes (HSG) in the course of human evolution are presumed to have enabled the adaptation of humans to new environments and new behaviors [1], [2], [3], [4], [5], [6], [7], [8], [9] but their specific physiological functions are often unknown. The human-specific CHRFAM7A gene is a case in point. First, it encodes a uniquely human and independently regulated subunit of the α7-nicotinic acetylcholine receptor (α7-nAChR) that regulates neurotransmitter function. Therefore, it is presumed to affect CNS function. When co-expressed with α7-nAChR, however, CHRFAM7A is a dominant negative regulator of neurotransmitter binding to, and activation of, the α7-nAChR, thereby potentially altering the central nervous system functions of α7-nAChR in its regulation of processes like cognition, memory, and mental health [10], [11], [12], [13], [14], [15], [16], [17], [18], [19].

But CHRFAM7A is also widely expressed in leukocytes and epithelial cells [11], [19], [20], [21], where it is presumed to regulate the powerful anti-inflammatory effects of α7-nAChR activation [22], [23], [24]. Because the activation of α7-nAChR is an obligatory intermediate for vagus nerve control of inflammation [25], CHRFAM7A in humans must therefore regulate the anti-inflammatory vagus nerve. If so, it raises the possibility that CHRFAM7A expression in peripheral tissues [20], [21] could be associated with human inflammatory disease like, for example, inflammatory bowel disease (IBDs).

IBDs have complex molecular etiologies of genetic, epigenetic, microbial, and environmental origin that present as highly heterogeneous episodes of gut inflammation [26], [27], making animal modeling difficult [28]. Accordingly, the response of IBDs to behavioral, dietary, and therapeutic interventions is often enigmatic, as exemplified by both protective and deleterious effects of nicotine and nicotine withdrawal on its remission, recurrence, and treatment [26], [27]. Here, we explored the possibility that a concomitant, but differentially regulated [20], [21] expression of the human-specific and pro-inflammatory CHRFAM7A gene and its anti-inflammatory α7-nAChR target [11], [19], [24], [25] (CHRNA7), could be implicated in IBD.

## 2 Materials and methods

### 2.1 Biopsies of inflammatory bowel disease and colon cancer

cDNAs in OriGene TissueScan Arrays from characterized biopsies of ulcerative colitis (CCRT101 and CCRT 102), Crohn's disease (CCRT101 and CCRT 102), or colon cancer (HCRT104) were used to assess gene expression in disease (N = 109) and control (N = 19) tissue biopsies. All characteristics of these specimens are available online with detailed clinical information, histology slides of each biopsy, and the quality control data for RNA isolation and cDNA preparations at California http://www.origene.com/qPCR/Tissue-qPCR-Arrays.aspx. The original de-identified tissues were collected from accredited medical institutions in the United States using IRB-approved protocols, selected by board-certified pathologists and then deposited into the OriGene tissue biorepository along with all of the available clinical data supporting the pathology diagnoses. The specific array plates used contained cDNA synthesized prepared from RNA extracted from these pathologist-verified tissues. The quantity of cDNA was normalized, first validated with ß-actin at OriGene and the findings replicated in the course of the gene expression studies described here. The Ct value of actin gene expression in each well was determined in our laboratories was highly consistent (average 20.89 cycles ± 0.1 (SEM, N = 135)) and the individual values from each well used to calculate relative gene expression in each biopsy using the actin primers, as specifically noted by the array manufacturer.

### 2.2 PCR, primers, and the conditions for CHRFAM7A, CHRNA7, and TBC1D3 analyses

The PCR reaction was performed in 50 μl containing 45 μl PCR blue mix (Invitrogen), 1 μl of each primer (10 μM), 300 ng cDNA, and 2 μl water. The cycling conditions were 94 °C for 4 min followed by 35 cycles of 94 °C for 30 s, 60 °C for 30 s, and 72 °C for 60 s, and a final extension at 72 °C for 5 min. Ten microliters of each PCR product was resolved on a 2% agarose gel and images were acquired using Alpha Innotech imaging system. Real-time qPCR was performed in a 25 μl reaction containing 12.5 μl 2 × CYBR Green PCR Master Mix (BioRad), 0.5 μl of each primer (10 μM), 1 μl cDNA, and 10.5 μl water. PCR cycling conditions were 95 °C for 10 min followed by 45 cycles of 94 °C for 25 s, 60 °C for 25 s, and 72 °C for 40 s. Primer efficiency for CHRFAM7A and CHRNA7 were 100% and 94%, respectively.

Expression of CHRNA7 and CHRFAM7A was normalized to that of β-actin using ΔΔCt method and primers provided by the manufacturer of tissue arrays. Primers for CHRFAM7A were designed to hybridize with the variant 1 transcript by selecting oligonucleotide sequences bridging shared CHRFAM7A and CHRNA7 sequences and therefore unique to CHRFAM7A and not available in CHRFAM7A or CHRNA7A alone.

**Table:** 

Sense:	5′-ATAGCTGCAAACTGCGATA-3′,
Anti-sense:	5′-CAGCGTACATCGATGTAGCAG-3′

Primers for CHRNA7 were designed to hybridize with both variant 1 and 2 transcripts of human CHRNA7 by selecting oligonucleotide sequences that are present in both variants of human CHRNA7 but absent from CHRFAM7A.

**Table:** 

Sense:	5′-ACATGCGCTGCTCGCCGGGA-3′,
Anti-sense:	5′-GATTGTAGTTCTTGACCAGCT-3′.

Primers for TBC1D3 were selected based on previously published findings [29]:

**Table:** 

Sense:	5′-GCATCGACCGGGACGTAAG-3′,
Anti-sense:	5′-CCTCCGGGTTGTACTCCTCAT-3′.

### 2.3 Analyses of gene expression

CHRFAM7A, CHRNA7, TBC1D3, and actin gene expression were measured as described above and normalized to that of β-actin or as indicated, to CHRNA7 expression, in each sample. The fold change in gene expression was calculated by ΔΔCt method and analyzed using the relative expression software tool (REST) for group-wise comparisons of relative expression [30]. The GraphPad program PRISM6 was used for preparation of figures.

## 3 Results and discussion

The characteristics of the commercially available gene expression arrays of human IBD and colon cancer used in these studies are publicly available http://www.origene.com/qPCR/Tissue-qPCR-Arrays.aspx and include information regarding gender, age, tissue of origin, case diagnosis from donor institutions, histological sections, and pathology verification reports. The latter also includes the percentage of mucosa/differentiation, lesion, and inflammation and are summarized in Table 1.

### 3.1 CHRFAM7A and the gene encoding human α7-nAChR (CHRNA7) are differentially expressed in IBD

**Fig. 1:**
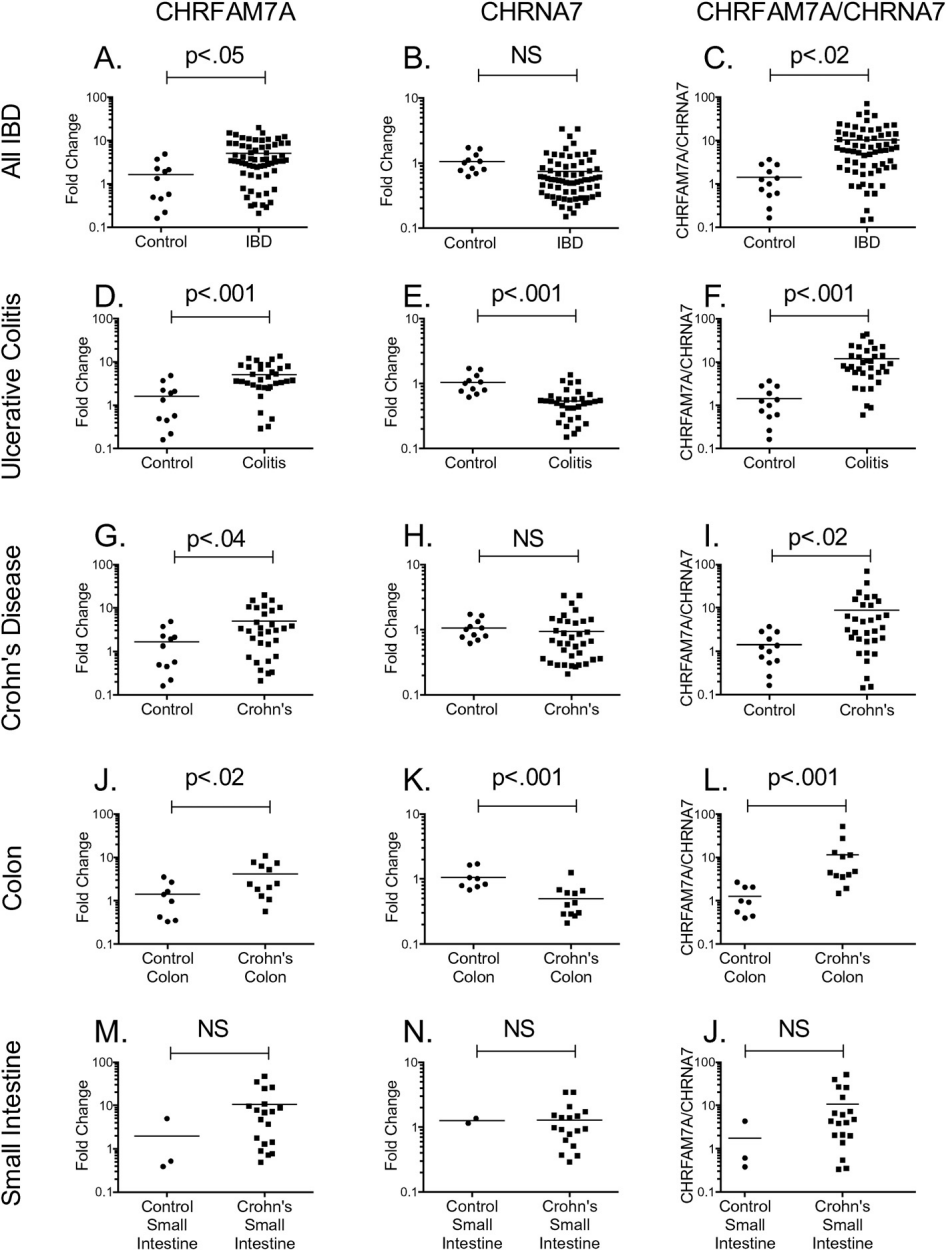
CHRFAM7A and CHRNA7 gene expression in inflammatory bowel disease (IBD). Quantitative RT-PCR was used to measure CHRFAM7A and CHRNA7 gene expression in intestinal biopsies from patients with IBD (panels *A*–*C*), ulcerative colitis (panels *D*–*F*), and Crohn's disease (panels G–J). Gene expression in each sample was normalized to that of actin or, as indicated, between each other to determine the CHRFAM7A/CHRNA7 ratio. Relative gene expression was compared to that in control biopsies using the ∆∆Ct method. Differences in gene expression between the control and disease biopsies were measured by REST [30] for group-wise comparisons and evaluated as either individual plates and after combination (shown).

Initially, quantitative RT-PCR of biopsies (N = 64) from patients with IBD suggested that there was only a small, albeit significant (p < 0.05) increase in CHRFAM7A gene expression (Fig. 1*A*) compared to biopsies from control tissue. In cDNA prepared from these same biopsies, there was no significant difference in CHRNA7 gene expression (Fig. 1*B*) although the ratio of CHRFAM7A/CHRNA7 gene expression (Fig. 1*C*) was different in IBD (p < 0.02) compared with controls.

In contrast, when we compared CHRFAM7A and CHRNA7 gene expression in the two distinct forms of disease represented in IBD (ulcerative colitis and Crohn's disease), a significant pattern emerged. First, colon biopsies from patients with ulcerative colitis (N = 33) confirmed the global increase in expression of CHRFAM7A (Fig. 1*D*), but now the analyses revealed that there was a concomitant and significant (p < 0.001) decrease in CHRNA7 expression in ulcerative colitis (Fig. 1*E*). These changes in ulcerative colitis were also highly significant (p < 0.001) when comparing CHRNA7 expression to that of CHRFAM7A (Fig. 1*F*). In Crohn's disease (N = 31), there was a small but significant (p < 0.04) increase in CHRFAM7A gene expression (Fig. 1*G*) but no significant change in CHRNA7 expression (Fig. 1*H*). As earlier, normalization of CHRFAM7A with CHRNA7 expression increased the significance of the difference (p < 0.02, Fig. 1*I*).

Ulcerative colitis affects colon and not small intestine but Crohn's disease can affect any portion of the gastrointestinal tract [26], [27]. In analyzing the source of biopsy **(**Fig. 1*J*, *K*, and *L*), we observed a significant (p < 0.02) up-regulation in CHRFAM7A gene expression in colon from patients with Crohn's disease (Fig. 1*J*), but there was also a concomitant and significant (p < 0.001) down-regulation in CHRNA7 (Fig. 1*K*) underscored by the significant change of CHRFAM7A when normalized with CHRNA7 (Fig. 1*K*). In small intestine biopsies of Crohn's disease, the change in CHRFAM7A (Fig. 1*M*), CHRNA7 (Fig. 1*N*) or in the ratio of CHRFAM7A to CHRNA7 (Fig. 10) was not significant.

### 3.2 The changes in CHRFAM7A and CHRNA7 are specific

**Fig. 2:**
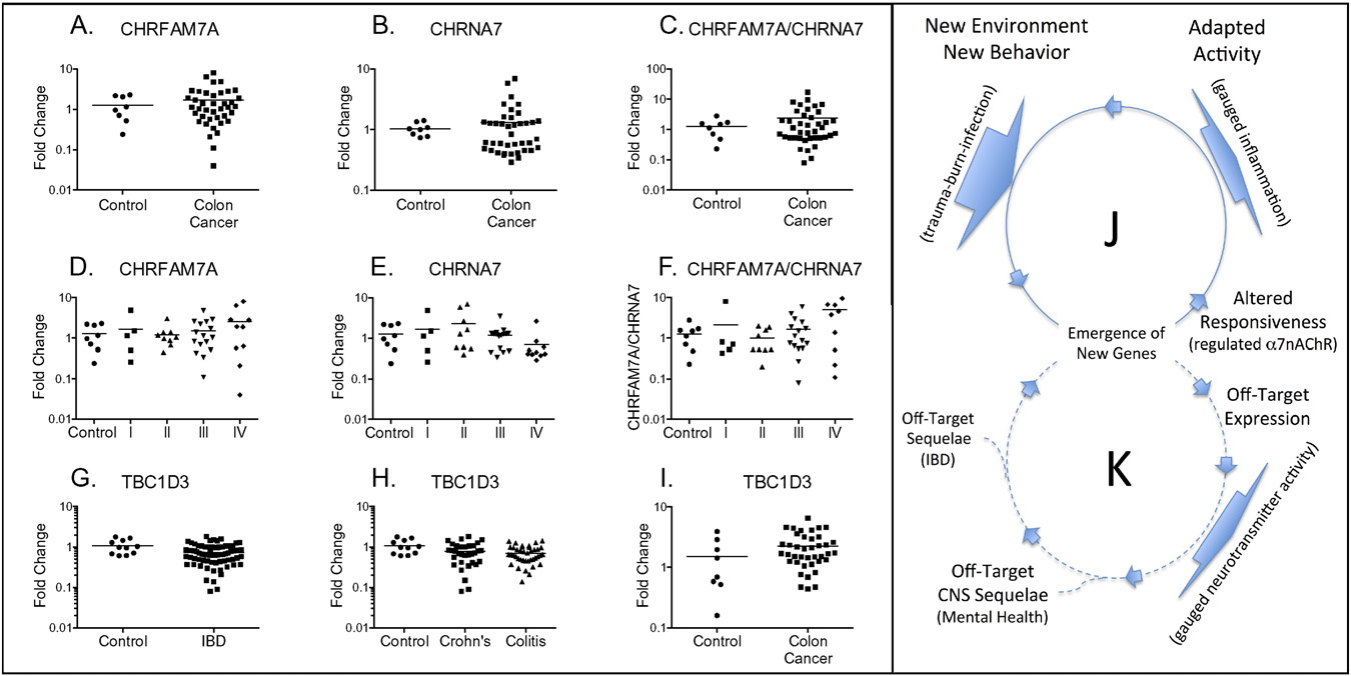
Changes in CHRFAM7A and CHRNA7 gene expression are specific for IBD. Quantitative RT-PCR was used to measure CHRFAM7A and CHRNA7 gene expression in biopsies of colon cancer (panels *A*–*F*) as all biopsies (panels *A*–*C*) or by colon cancer stage (panels *D*–*F*). Gene expression in each sample was normalized to that of actin or, as indicated, between CHRFAM7A and CHRNA7 using the ∆ Ct method. Relative gene expression was then compared to that in control biopsies using the ∆∆Ct method. Differences in gene expression between the control and disease biopsies were measured by REST [30] for group-wise comparisons and no differences were found to be significant at p < 0.05. Expression of the human-specific gene TBCD1 from all IBD samples (panel *G*), in Crohn's and ulcerative colitis (panel *H*) or in colon cancer (panel *I*) were also unchanged. As illustrated in panel *J*, new genes emerge in new environments to adapt to new behaviors like bipedal behavior (trauma) and the harnessing of fire (burn/sepsis) and alter responsiveness. Off-target effects (panel *K*) for example regulating α7-nAChR activity in neurons might prove even more important than the original pro-inflammatory selection but the sequelae for human disease tied to original (inflammation) and unanticipated (mental health) effects of gene expression.

We used two approaches to establish specificity of differential expression in diseased colon. First, we evaluated the expression of CHRFAM7A (Fig. 2*A* and *D*), CHRNA7 (Fig. 2*B* and *E*), and CHRFAM7A-to-CHRNA7 ratio (Fig. 2*C* and *F*) in colon cancer biopsies. No differences were detected when all colon cancer biopsies were evaluated together (Fig. 1*A*, *B*, *C*) or when analyzed according to the stage of disease (Fig. 1*D*, *E*, F). Second, we evaluated the expression of a second human-specific gene called TBC1D3, that is associated with macro-pinocytosis and epidermal growth factor signaling [29]. There were no differences in IBD, no differences in either ulcerative colitis or Crohn's disease when examined separately. There were also no differences in gene expression of TBC1D3 in biopsies from colon cancer (Fig. 2*G*, *H*, *I*). This concordance of CHRFAM7A and CHRNA7 expression in colon cancer is also evident in curated public databases like the Cancer Genome Atlas (www.cbioportal.org), which enables mining gene expression patterns in different epithelial cancers. Correlations between CHRFAM7A and CHRNA7 in these databases are > 0.87 (Pearson) and > 0.77 (Spearman) in uterine, stomach, and colorectal cancers). Unfortunately, no analogous public databases with RNAseq data exist for inflammatory bowel disease, Crohn's disease, or ulcerative colitis, although several studies have evaluated whole genome gene expression [31], [32], [33] and found changes in, and effects of, traditional inflammatory products like TNF and HMBG1 [34], [35].

In 2011, Cooper and Kehrer-Sawatzki [8] reported that new human genes [2], [3], [5], [7], [8], [9] are over-represented among genes tied to complex human disease and more recently [36] described how newly evolved human genes can drive gene interaction networks associated with critical phenotypes. It is in this vein that the results presented here suggest that the up-regulation of pro-inflammatory CHRFAM7A in humans could exacerbate the down-regulation of anti-inflammatory α7-nAChR in IBD. If so, it is interesting to speculate that this pro-inflammatory effect of CHRFAM7A expression is an “off-target” contributor to human IBD that arose as a function of adaptation (Fig. 2*J*, *K*). In this paradigm, a human-specific gene like CHRFAM7A could have originally arisen as an evolutionary pro-inflammatory and adaptative response to newly emerging human behaviors like bipedal walking (trauma) or the harnessing of fire (burn injury) but retained for CNS activities regulating neurotransmitter activity. Interestingly, human-specific responses in gene expression after trauma, burn, and infection have been previously described [37], although they remain controversial largely because of their implications to animal modeling of human injury [38], [39]. Ironically, the putative adaptive pro-inflammatory origin of a hominid gene like CHRFAM7A may ultimately be ancillary to its physiological significance to human speciation because CHRFAM7A in the brain is tied to regulating α7-nAChR, a ligand-gated neurotransmitter channel that itself regulates human cognition, attention, memory, and mental health. In this model, the up-regulation of CHRFAM7A in peripheral tissues of modern humans could reflect vestigial pro-inflammatory activity. Such a possible paradigm underscores the importance of understanding the role of human evolution in the etiology of human disease, the role of HSGs, and ultimately, their function when modeling human disease.

On a final note, the differential expression of the CHRFAM7A human-specific gene in a prototypic human disease like IBD underscores the importance of better understanding the contribution of this class of genes to the onset, development, and progression human disease when diseases are modeled in experimental animals. With newly emerged human-specific genes like ARHGAP11B promoting neocortex expansion *in vivo* [40], c20orf203 eliciting differential gene function [41], human-specific defensins conferring differential resistance to infection [42], and CD33 providing cognitive protection [43], it is critical to understand the possible contributions of newly evolved gene interaction networks to human disease when they differ in humans from all other species and create unique phenotypes [36].

## Author contributions

Conceived of experiments and wrote first drafts of manuscript (AB), designed PCR and validated qPCR (XD), assisted in interpretation of data and assisted in preparation of brief report (RC, BE, and TC).

## Conflict of interest statement

The authors have declared that no conflict of interest exists.

## Figures and Tables

**Table 1:** Tissue biopsies.

	Ulcerative colitis	Crohn's disease	Colon cancer			
*Array ID*	CCRT101/102	CCRT101/102	HCRT104			
*Biopsies studied*	44	42	48			
Normal	11		8			
Disease	33	31	40			
*Control biopsies*						
Location of lesion	Colon 8		Colon 8			
	Small Intestine 3					
*Gender*						
Male	7		2			
Female	4		6			
Age (years)	54 (26–89)		78 (60–89)			
Male	56 (26–89)		82 (81–82)			
Female	50 (29–70)		77 (60–89)			
% Mucosa	48% (10–90)		N/A			
Male	50% (20–85)		N/A			
Female	44% (10–90)		N/A			
*Disease biopsies*			Tissue	Stage		N
Location of lesion	Colon 33	Colon 13	Colon	l		5
	Small Intestine 0	Small Intestine 18	Colon	ll		9
			Colon	lll		16
			Colon	lV		10
*Gender*						
Male	21	14	16			
Female	12	17	24			
Age (years)	39 (22–76)	38 (19–65)	68 (21–89)			
Male	38 (22–72)	40 (20–64)	65 (21–82)			
Female	45 (26–76)	36 (19–65)	70 (45–89)			
			Well	Moderate	Poor	Un-diff.
% Mucosa/differentiation*	43% (10–100)	38 (0–00)	10	19	6	5
Male	43% (10–100)	41 (0–80)	7	11	4	2
Female	43% (10–95)	35 (10–90)	3	8	2	3
*Pathology*						
Lesion/tumor (%)	100		71% (25–95%)			
Male	100		69.9 (25–95)			
Female	100		73.7 (40–90)			
Hypercellular stroma (%)	N/A		16% (0–55%)			
Male	N/A		17.3 (0–55%)			
Female	N/A		14.4 (0–35%)			
Hypocellular Stroma (%)	N/A		1.95% (0–28%)			
Male	N/A		2.7 (0–28%)			
Female	N/A		0.9 (0–10%)			
Necrosis (%)	0		4.9% (0–40%)			
Male	0		3.6% (0–40%)			
Female	0		6.6 (0–20%)			
